# Cancer-Associated Glycosphingolipids as Tumor Markers and Targets for Cancer Immunotherapy

**DOI:** 10.3390/ijms22116145

**Published:** 2021-06-07

**Authors:** Sophie Groux-Degroote, Philippe Delannoy

**Affiliations:** CNRS, UMR 8576—UGSF—Unité de Glycobiologie Structurale et Fonctionnelle, University of Lille, F-59000 Lille, France; philippe.delannoy@univ-lille.fr

**Keywords:** ganglioside, cancer, glycosyltransferase, immunotherapy, transcriptional regulation, Golgi apparatus

## Abstract

Aberrant expression of glycosphingolipids is a hallmark of cancer cells and is associated with their malignant properties. Disialylated gangliosides GD2 and GD3 are considered as markers of neuroectoderm origin in tumors, whereas fucosyl-GM1 is expressed in very few normal tissues but overexpressed in a variety of cancers, especially in small cell lung carcinoma. These gangliosides are absent in most normal adult tissues, making them targets of interest in immuno-oncology. Passive and active immunotherapy strategies have been developed, and have shown promising results in clinical trials. In this review, we summarized the current knowledge on GD2, GD3, and fucosyl-GM1 expression in health and cancer, their biosynthesis pathways in the Golgi apparatus, and their biological roles. We described how their overexpression can affect intracellular signaling pathways, increasing the malignant phenotypes of cancer cells, including their metastatic potential and invasiveness. Finally, the different strategies used to target these tumor-associated gangliosides for immunotherapy were discussed, including the use and development of monoclonal antibodies, vaccines, immune system modulators, and immune effector-cell therapy, with a special focus on adoptive cellular therapy with T cells engineered to express chimeric antigen receptors.

## 1. Biosynthesis and Expression of Cancer-Associated Gangliosides

Gangliosides are cell surface glycosphingolipids that play important functional roles in cell-cell recognition, cell adhesion, and signal transduction. Their carbohydrate moiety is synthesized in the Golgi apparatus by the sequential action of different glycosyltransferases. Modifications in expression patterns of gangliosides during development or diseases can be largely attributed to changes of the expression of glycosyltransferases involved in their synthesis and that are spatiotemporally regulated both at the transcriptional and post-translational levels [1]. Regulation at the transcriptional level includes transcription factors (TF), but also epigenetic modifications and can explain most of the glycolipid composition changes observed during development and cancer [2,3].

Although gangliosides are mostly expressed in neural tissue, abundant expression of specific gangliosides has been observed in some tumors. A characteristic feature of small cell lung carcinoma (SCLC) is the aberrant and abundant expression of ganglioside Fuc-GM1 (fucosylated monosialotetrahexosylganglioside or IV^2^FucII^3^Neu5AcGg_4_Cer), whereas the ectopic expression of b-series gangliosides and their *O*-acetylated derivatives is associated with cancers of neuro-ectoderm origin.

### 1.1. Biosynthesis Pathways of Cancer-Associated Gangliosides

**Figure 1:**
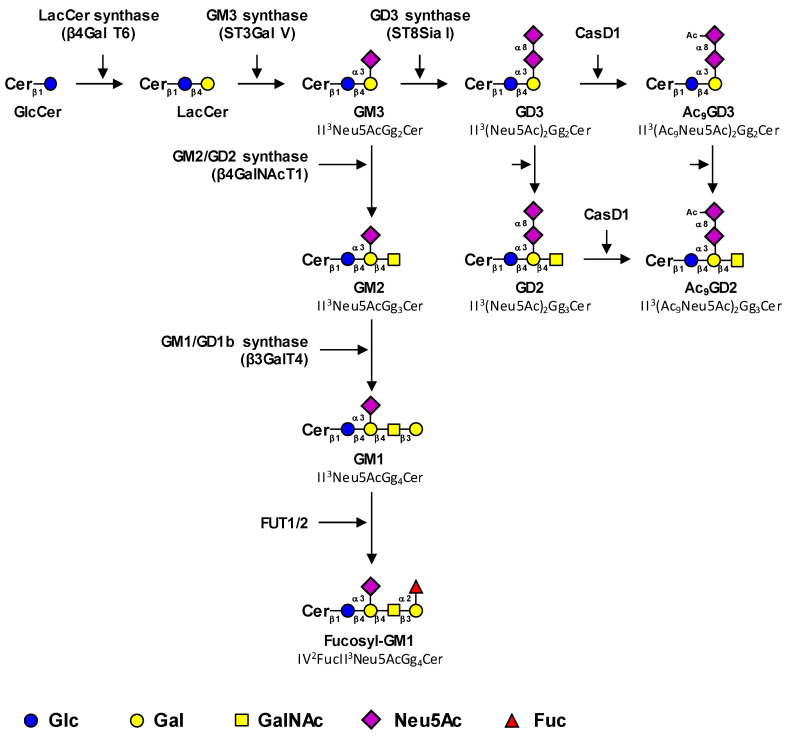
Proposed biosynthesis pathway for tumor-associated gangliosides. Tumor-associated gangliosides are synthesized from lactosyl-ceramide (LacCer) by the action of the GM3 synthase ST3Gal V that transfers a first sialic acid residue to form GM3 (precursor of the a-series gangliosides). The elongation of GM3 is performed by the sequential action of the β4GalNAc T1 and β3Gal T4 to form GM1, which is further fucosylated by the α1,2-fucosyltransferases FUT1 and FUT2. Alternatively, the action of the GD3 synthase ST8Sia I converts GM3 in GD3, the precursor of the b-series gangliosides, which is the substrate of the β4GalNAc T1 that forms GD2. Finally, GD3 and GD2 can be acetylated by CASD1 sialate O-acetyl-transferase on the C9 position of sialic acids to form 9-*O*-acetylated GD3 (9-*O*AcGD3) and 9-*O*-acetylated GD2 (9-*O*AcGD2), respectively. It has also been proposed that O-acetylated GD3 can be formed by the transfer of CMP-Neu5,9Ac_2_ on GM3 by the GD3 synthase. The effects of β4GalNAc T1 and β3Gal T4 on OAcGD3 and OAcGD2, respectively, are currently unknown.

The biosynthesis of tumor-associated gangliosides starts from lactosylceramide (LacCer, Gg_2_Cer) by the transfer in the Golgi apparatus of a first sialic acid residue catalyzed by the GM3 synthase ST3Gal V to form GM3 (II^3^Neu5AcGg_2_Cer) (Figure 1). The GM3 synthase, encoded by the *ST3GAL5* gene, is the only sialyltransferase that uses LacCer as an acceptor substrate [4]. However, it was demonstrated that the product of the *ST3GAL5* gene was also able to synthesize GM4 from galactosylceramide, specifically expressed in the brain and kidneys [5]. Three GM3 synthase isoforms with different N-terminal cytoplasmic tail lengths were characterized [6].

Little is known about *ST3GAL5* gene regulation. However, it was shown that Zeb1, a transcription factor associated to epithelial-mesenchymal transition (EMT), induces the expression of the GM3 synthase via the binding of Zeb1 to the *St3gal5* promoter and the suppression of microRNA-mediated repression of *St3gal5* [7].

Thereafter, GM3 can be elongated by the sequential action of the GM2/GD2 synthase β4GalNAcT1 and the GM1/GD1b synthase β3GalT4 to form GM1 (Neu5AcGg_4_Cer), which belongs to the a-series gangliosides (Figure 1) [8,9]. The β4GalNAcT1 is active on all series of gangliosides and converts LacCer, GM3, GD3, and GT3 (the precursors of the 0, a, b and c-series gangliosides, respectively) into GA2, GM2, GD2, and GT2, respectively (Figure 1) [10,11]. Similarly, the β3GalT4 equally uses GM2 and GD2 as acceptor substrates [10]. Both enzymes can form functional complexes in the trans-Golgi network, which may optimize complex glycolipid biosynthesis by a channeling effect [12].

GM1 can be further fucosylated by α1,2-fucosyltransferases to form fucosyl-GM1 (Figure 1). In humans, two α1,2-fucosyltransferases, the H and Se enzymes encoded by *FUT1* and *FUT2* genes respectively, are potentially involved in Fuc-GM1 biosynthesis. These GDP-l-Fuc: galactoside α1,2-l-fucosyltransferases belong to the Cazy superfamily of GT11 (EC 2.4.1.69) and are involved in the transfer of l-Fucose (Fuc) in α1,2-linkage onto the terminal galactose of (N-acetyl)lactosamine units [13]. Individuals of histoblood-group “O” express the H antigen under control of the *FUT1* gene on red cells and on the vascular endothelium, whereas the H antigen is under control of the *FUT2* gene in exocrine secretions. Both enzymes are involved in the biosynthesis of the Lewis b (Fucα1-2Galβ1-3[Fucα1-4]GlcNAc) and Lewis Y (Fucα1-2Galβ1-4[Fucα1-3]GlcNAc) antigens on glycoproteins and glycolipids in endoderm-derived epithelial tissues, such as the digestive tract and salivary glands [14].

Studies performed in different Fuc-GM1-positive small lung cancer cell lines showed that both *FUT1* and *FUT2* genes were expressed and both fucosyltransferases FUT1 and FUT2 were potentially able to α1,2-fucosylate GM1 [15]. Overexpression of both genes increased the Fuc-GM1 expression in SCLC cells. However, three out of four cell lines used in this study exhibited mutations in the *FUT2* coding region, resulting in the loss of FUT2 enzymatic activity, suggesting that FUT1 is the biologically relevant gene for Fuc-GM1 biosynthesis in SCLC cell lines. Interestingly, the use of *FUT1* and *FUT2* knock-out mice showed that both Fuc-GM1 and Fuc-GA1 totally disappeared from the antrum, cecum, and colon in the gastro-intestinal tract of *FUT2*-null mice, whereas the levels of both glycolipids were normal in *FUT1*-null and wild-type mice. In parallel, GM1 and GA1, which are the precursors of Fuc-GM1 and Fuc-GA1, accumulated in the tissues of *FUT2*-KO mice, confirming that FUT2 is preferentially involved in the fucosylation of GA1 and GM1 in murine tissues [16].

The aberrant expression of Fuc-GM1 in a restricted variety of tumors, and the role of this glycolipid in cancer, remains to be elucidated. Whether a tumor expresses Fuc-GM1 or, rather, b-series gangliosides such as GD3 and GD2 depends mostly on the glycosyltransferase expression pattern. In the SK-LC-17 SCLC cell line, the co-transfection of GM1 synthase cDNA with *FUT1* or *FUT2* increased Fuc-GM1 expression, whereas the co-transfection of GD3 synthase cDNA with *FUT1* or *FUT2* reduced the expression levels of Fuc-GM1. The regulation of genes encoding key glycosyltransferases that allow a switch from the a-series to the b-series gangliosides, as well as *FUT1* and *FUT2* regulation, is a major element to understand the specific cancer-associated ganglioside expression.

Alternatively, the action of the GD3 synthase ST8Sia I converts GM3 into GD3, which is the substrate of GD2 synthase β4GalNAc T1 that forms GD2. ST8Sia I, encoded by the *ST8SIA1* gene, is the key enzyme that allows a switch from the simple (0- and a-series) gangliosides to complex highly sialylated gangliosides from the b- and c-series. The prevalent expression of GD3 or GD2 is mainly controlled by the relative expression of glycosyltransferases involved in their synthesis, GD3 synthase and GD2 synthase respectively. For example, the high GD2/GD3 ratios observed in neuroblastoma result from both a low expression of GD3 synthase and a high expression of GD2 synthase, whereas the opposite ratios and expression levels are observed in melanoma, in which GD3 is the most expressed [17,18]. The expression level of GD3 synthase and/or GD2 synthase mRNA are often augmented in cancers compared to healthy controls [19,20] and can be considered as malignancy markers associated with histopathological grading and/or prognosis. The promoter region of the *ST8SIA1* gene has been described in melanoma [21,22], glioblastoma [23], and breast cancer cell lines [24], showing a unique transcript with transcription start sites located 450 to 690 bp upstream of the initiation codon on the first exon. In glioblastoma cells, analysis of the *ST8SIA1* promoter has shown the role of AREB6 and Elk-1 transcription factors in GD3 synthase gene transcription [23]. EMSA and mutagenesis experiments have also demonstrated the key role of the nuclear factor-κB (NFκB) in activating the expression of the GD3 synthase in SK-MEL-2 melanoma [22] and breast cancer cells [24]. In addition, in estrogen-receptor-positive breast cancer cells, estradiol prevents NFκB binding to the *ST8SIA1* promoter by inhibiting nuclear translocation of NFκB subunits [24].

Finally, the sialic acid residues of GD3 and GD2 can be *O*-acetylated, mainly in position nine. To date, CASD1 (CAS1 Domain-Containing Protein 1) is the only sialate-*O*-acetyl-transferase (SOAT) characterized in humans. Data from Baumann and co-workers suggested that CASD1 was involved in 9-*O*-acetylation of sialic acids in human HAP-1 cells and resulted in increased 9-*O*AcGD3 expression [25]. In parallel, CASD1-deficient mice exhibited a complete loss of *O*-acetylation of sialic acid on the murine erythrocyte cell surface [26]. Nevertheless, even if CASD1 seems to play a key role in the *O*-acetylation process, the *O*-acetylation pathways for gangliosides remain rather unclear, especially for species other than OAcGD3.

### 1.2. Expression of Cancer-Associated Gangliosides in Healthy Cells/Tissues

The general knowledge states that disialogangliosides (also named complex gangliosides) enhance tumor cell phenotypes, whereas monosialylgangliosides tend to suppress them [27]. GD3 (II^3^(Neu5Ac)_2_Gg_2_Cer) and GD2 (II^3^(Neu5Ac)_2_Gg_3_Cer) are the most studied disialogangliosides; they belong to the b-series gangliosides with two sialic acid residues that are linked to lactosylceramide.

Complex gangliosides show high expression and play important roles during developmental stages, but their expression is low or lost in non-neural healthy adult tissues. GD3 and GD2 are predominantly expressed in the brain and in peripheral nerve tissues, but also on lymphocytes [28]. The expression of *O*-acetylated gangliosides was mostly studied in central nervous system (CNS) wherein they could play key roles during development. *O*AcGD3 expression was characterized during development in the central and peripheral nerve systems, and in retina in rats [29]. High levels of both *O*AcGD3 and *O*AcGT3 are expressed in the fetal brain in different species and rapidly decrease after birth [30]. *O*AcGD3 and *O*AcGT3 are believed to play a role in neurite extension during CNS development [31]. *O*AcGD3 may promote the extension of growth cones and neuronal cell differentiation by interacting with specific proteins, as described for other types of gangliosides involved in well-studied biological functions. Disialoganglioside GD3 and its *O*-acetylated derivatives 9-*O*AcGD3 and 7-*O*AcGD3 have been identified at the cell surface in human T lymphocytes of the peripheral blood system and on human tonsillar lymphocytes and are considered as markers of their proliferation after activation [32].

Fuc-GM1 was primarily identified in the CNS. Fuc-GM1 expression is developmentally regulated and is normally expressed in a subset of peripheral sensory neurons and dorsal root ganglia [33,34]. The biological functions of α1,2-fucosylated glycolipids remain largely unknown. Studies in Neuro-2 neuroblastoma cells suggested that the expression α1,2-fucosyltransferase, and the subsequent formation of fucosyl-GM1, modulate axonal outgrowth and the response of neuronal cells to signal control axonal extension [35].

## 2. Expression and Roles of Cancer-Associated Ganglioside in Malignant Properties

### 2.1. Expression of Cancer-Associated Gangliosides in Cancers

#### 2.1.1. Expression of b-Series Gangliosides and Their *O*-Acetylated Derivatives in Cancer

Both GD2 and GD3 are overexpressed in neuroectoderm-derived tumors at different rates depending on the cancer type and are considered as markers in melanoma [36], glioblastoma [37], pediatric T lymphoblastic malignancies [38], soft tissues sarcoma [39,40], breast cancer [41], lung cancer [42], and neuroblastoma [43]. GD3 and GD2 can be shed from the tumors and released into circulation. GD2 appears to be a specific and sensitive biomarker for high-risk/high-stage neuroblastoma with a potential use as a diagnostic or prognostic biomarker [44]. The mechanisms involved in GD3- and GD2-modulation of malignant cell properties have been extensively studied, especially in melanoma, neuroblastoma, and glioblastoma, but also in sarcoma and breast cancer, and are described in Section 2.2 of this review.

Sialic acid *O*-acetylation is a developmentally regulated modification of gangliosides implicated in cell proliferation, neural cell migration, and peripheral nerve regeneration, but also in tumor progression. *O*-acetylated b-series gangliosides, especially OAcGD3OAcGD2, are also considered as tumor-associated carbohydrate antigens in neuro-ectoderm derived cancers. These *O*-acetylated gangliosides are getting more and more attention from the scientific community, especially because their expression pattern is even more restricted to healthy tissues compared with their non-acetylated forms.

Immunological tools (monoclonal antibodies (mAb) D1.1 and JONES) allowed for the characterization of *O*AcGD3 in melanoma cells and tissues more than 30 years ago [45]. *O*AcGD3 is therefore the best-characterized and most studied *O*-acetylated ganglioside [46,47].

High levels of *O*AcGD3 were detected in several types of cancer, especially melanoma, but also glioblastoma, childhood acute lymphoblastic leukaemia, and in lung cancer cells [48,49,50]. Analytical techniques allowed for the identification of 9-*O*AcGD3 as the major *O*AcGD3 species in melanoma tissues and cells [51,52]. *O*AcGD3 is also detected in other neuro-ectoderm derived cancers, such as neuroblastoma [53], gliosarcoma [54], and breast cancer [55]. 9-*O*AcGD3 expression is modified in benign and atypical proliferative lesions and carcinomas of the human breast and increased in basal cell carcinoma tissues compared with the skin that surrounds cancer cells [56,57].

Enhanced levels of *O*AcGD3 have been reported in childhood lymphoblastic leukemia, both in lymphoblasts of patients and in leukemic cell lines, in comparison with normal cells [49]. Other gangliosides derived from GD3, such as GD2 or GT3, were also described to be *O*-acetylated. *O*AcGT3 expression was detected in gliosarcoma tissues, cultured glial cells, and breast cancer cells [52,54,55,58].

*O*-acetylated GD2 has recently received significant attention as a novel antigen to target GD2-positive cancers. *O*AcGD2 is highly expressed by GD2-positive tumors such as sarcomas, neuroblastomas, gliomas, and in small cell lung cancer and breast cancer cells [59,60]. GD2 has been considered for more than two decades as a tumor-associated antigen and a highly valuable therapeutic target in these cancers. Consequently, anti-GD2 mAb have been developed to treat these cancers, and chimeric mAb ch14.18 has become the benchmark for neuroblastoma therapies [61]. Unfortunately, GD2 is expressed after birth in the normal brain and in peripheral nerves, which limits the use of anti-GD2 mAbs in cancer immunotherapy (this issue is discussed in Section 3). A focus on *O*AcGD2 as a marker and target of interest in cancer has been allowed by the recent development of tools, i.e., mAbs that specifically recognize the *O*-acetylated GD2 species, such as 8B6 mAb, and analytical techniques that do not induce the loss of the alkali labile acetyl group. Preliminary data on the use of anti-*O*AcGD2 mAbs provide an important rationale for the clinical application of c.8B6 in patients with high-risk neuroblastoma [62].

Interestingly, *O*AcGD2 is not detected in normal brain tissue nor in peripheral nerves but is overexpressed in most GD2-positive cancers. The anti-*O*AcGD2 8B6 mAb strongly reacted in numerous malignant tissues, and especially with neuroblastoma and melanoma tissues and cell lines [63,64], but also in glioblastoma multiforme (GBM) biopsies, another GD2-positive cancer [65]. Several studies reported the expression and role of ganglioside GD2 in breast carcinomas, in which GD2 is considered as a marker of breast carcinoma stem cells capable of initiating tumors at a higher frequency than GD2-negative cells [41,66]. Importantly, these studies were performed using the anti-GD2 mAb 14G2a that cross-reacts with *O*AcGD2 ganglioside. The expression and role of *O*AcGD2 in breast cancer stem cells still remains to be investigated.

The analysis of ganglioside *O*-acetylation and expression patterns in more tissues, in physiological and pathological conditions, is now required to provide a more global view on the fine structures of *O*-acetylated gangliosides and identify new species that could be used as targets for cancer immunotherapy.

#### 2.1.2. Expression of Fuc-GM1 in Cancer

Accumulation of Fuc-GM1 and α-galactosyl-α-fucosyl-GM1 (IV^3^GalIV^2^FucII^3^Neu5AcGg_4_Cer) was reported in precancerous livers of rats fed with the carcinogen N-2-acetylaminofluorene and could play a role in the development of hepatoma. This accumulation of α1,2—fucosylated glycolipids was linked to an increased α1,2-fucosyltransferase activity toward GM1 and asialo-GM1 in liver extracts from rats fed with N-2-acetylaminofluorene compared with extracts from control group rat livers [67]. Numerous mAbs that specifically recognize Fuc-GM1 have been generated, allowing for the identification of this fucosylated ganglioside in tissues and in serum samples from cancer patients [68].

Since the 1990′s, aberrant Fuc-GM1 expression has been associated with SCLC, a subtype of lung cancer. Lung cancer is the leading cause of cancer-related death worldwide, with approximately 1.5 million deaths in 2012. SCLC comprises about 15% of lung cancer cases and is usually associated with a poor prognosis. The general five-year survival rate for people with SCLC depends on several factors, the most significant of which being the stage of disease. For localized SCLC, the overall five-year survival rate is 27%. If the cancer has spread outside of the lung to nearby areas or to distant parts of the body, the five-year survival rates are 16% and 3%, respectively.

Numerous studies revealed that a large percentage of SCLC tumors express very high levels of Fuc-GM1 (19 of 21 cases), whereas only 20% of squamous epithelial cell lung cancers and one of five large cell lung cancer specimens were positive. Lung adenocarcinoma and bronchial carcinoma were all negative for Fuc-GM1 expression. No Fuc-GM1 was detected in normal lung and bronchus, but staining was observed with occasional scattered expression on cells in the spleen, thymus, small intestine, and pancreas. All other normal tissues tested were negative [69]. Importantly, Fuc-GM1 was more frequently and strongly expressed in SCLC tumors than GD3, another TACA overexpressed in tumors, and seemed to be the most relevant ganglioside target for immunotherapy in SCLC [70]. 

Glycan microarray analysis was performed to identify potential TACA-binding antibodies in the serum from patients with HCC (hepatocellular carcinoma) that could be used as markers to distinguish between patients with HCC and healthy individuals [71]. Differences in serum antibody levels between patients with HCC and healthy individuals were highlighted, with antibody levels for 7 of 58 glycans that tested significantly higher in cancer patients, including antibodies against Fuc-GM1 and another glycolipid from the globo-series, disialyl-Gb5. This study suggested that Fuc-GM1 could be a marker for HCC. Antibodies of IgM and IgG classes reactive to Fuc-GM1 were also detected in sera of patients with differentiated thyroid cancer but did not provide any diagnostic or prognostic value [72].

### 2.2. Roles of Gangliosides in Malignant Properties of Cancer Cells

Gangliosides can influence tumor progression in multiple ways. First, gangliosides expressed at the cell surface, or shed from the cells into the circulation, can act as immunosuppressors, as typically observed for the suppression of T lymphocytes and dendritic cells [73]. Gangliosides, such as GD3, GD2/GM2, and GD1a, promote tumor-associated angiogenesis by increasing VEGF (vascular endothelial growth factor)-induced cell proliferation and migration [74,75]. Multiple studies also highlighted the regulation of cell adhesion/motility/metastasis by gangliosides. These ganglioside-mediated effects involve glycolipid-enriched domain (lipid rafts), specific raft-associated transducer proteins, and the activation of downstream signaling pathways [76]. Gangliosides can also generate metabolites, such as ceramide, ceramide-phosphate, and sphingosine, that modulate cancer cell properties [77].

#### 2.2.1. Biological Roles of b-Series Gangliosides and Their *O*-Acetylated Derivatives in Cancer

The role of disialogangliosides GD3 and GD2 in the proliferative and invasive capacities of tumor cells was primarily studied in tumors of neuro-ectoderm origin such as neuroblastomas, gliomas, and melanomas. For example, a decreased expression of GD3 in hamster melanoma cells led to a marked decrease in tumor growth and anti-GD3 mAb suppressed melanoma cell growth both in vitro and in vivo [78,79]. Similarly, the inhibition of GD3 expression in rat neuroblastoma cells reduced cell migration and metastatic potential in nude mice [80]. In parallel, the overexpression of GD3 synthase increased tumorigenicity and invasion of rat glioma cells, whereas anti-GD3 mAb specifically inhibited tumor growth [81,82]. In parallel, a large set of evidence suggested that GD3 and GD2 play a central role in malignancy by modulating receptor tyrosine kinase (RTK) signaling [83]. For example, a GD3-deficient mutant of the SK-Mel-28 melanoma cell line that only expresses a-series gangliosides showed decreased proliferation and motility, the proliferation and migration capacities of which being restored by transfection of GD3 synthase cDNA [36,84]. Thus, the binding of GD3 synthase-transfected cells to collagen type I leads to the phosphorylation of two focal adhesion proteins, paxillin and FAK, as well as the activation of the integrin-linked kinase-Akt (IKL-Akt) signaling pathway [2].

In MDA-MB-231 breast cancer cells, the expression of the GD3 synthase is associated with an increased proliferation in the absence of growth factors [85]. The GD3 synthase expression in MDA-MB-231 cells also enhanced tumor growth in severe combined immune-deficient (SCID) mice. In these cells, GD3 synthase expression induced the accumulation of GD3 and GD2 together with the acquisition of a proliferative phenotype under serum-free conditions due to the constitutive activation of the c-Met receptor, activating phosphoinositide 3-kinase (PI3K)/Akt and extracellular signal-regulated kinase (Erk)/mitogen-activated protein kinase (MAPK) pathways [86]. In parallel, the decrease of GD2 expression by GM2/GD2 synthase silencing reduced c-Met activation, and anti-GD2 mAb competition assays also inhibited cell proliferation and c-Met phosphorylation, demonstrating the involvement of GD2 in MDA-MB-231 cell proliferation through c-Met constitutive activation [66]. GD2 was also identified as a new specific cell-surface marker of breast cancer stem cells (CSCs) capable of forming mammospheres and initiating tumors [41]. The reduction of GD2 expression by GD3 synthase knockdown reduced mammosphere formation and completely inhibited tumor formation in vivo, changing the phenotype from CSC to non-CSC [41,87]. Moreover, the induction of EMT in transformed human mammary epithelial cells dramatically increased the GD3 synthase as well as GD2 expression, whereas the inhibition of the GD3 synthase compromised EMT initiation and maintenance and prevented metastasis [88].

In SCLC, the expression of GD2 is sufficient to increase cell growth and invasion [39]. In parallel, inhibition of GD2 expression reduced SCLC cell proliferation in vitro and tumor growth in SCID mice [89]. Competition assays with anti-GD2 mAb led to marked growth suppression and induced apoptosis of SCLC cells by activation of Erk/MAPK and p38/MAPK signaling pathways [42,90]. GD2-positive lung cells treated with anti-GD2 mAb undergo anoikis through the conformational changes of integrin molecules and subsequent FAK dephosphorylation, Erk, and p38 activation [90]. In pancreatic ductal adenocarcinoma, the most common pancreatic cancer, the GD3 level is very low despite the high sialylation status of that cancer. Interestingly, studies of pancreatic cancer cells transfected with GD3 synthase showed that GD3 expression induced cell cycle arrest and disruption of the integrin-β1-mediated anchorage, inhibited angiogenesis, and thereby induced apoptosis [91].

In contrast to GD3 and GD2, very little is known concerning the role of *O*-acetylated forms of disialogangliosides in cancers. An opposite effect of GD3 and *O*AcGD3 gangliosides has been described in acute lymphoblastic leukemia (ALL). Both GD3 and *O*AcGD3 were localized in the mitochondria outer membrane from patient lymphoblasts. The addition of exogenous GD3 induced lymphoblast apoptosis, whereas 9-*O*AcGD3 did not. GD3 caused the depolarization of mitochondrial membrane potential, the release of cytochrome c, and increased caspase 9 and caspase 3 activities, whereas 9-*O*AcGD3 failed to induce similar effects [56]. The protective effect of *O*AcGD3 from apoptosis was confirmed in Jurkat and in glioblastoma cells [48,92].

The anti-*O*AcGD2 chimeric mAb c.8B6 targeting *O*AcGD2, the other major *O*-acetylated ganglioside in cancer, significantly inhibited glioblastoma cell proliferation both in vitro and in vivo. It also induced CDC (complement-dependent cytotoxicity) and ADCC (antibody-dependent cell cytotoxicity) in vitro against *O*AcGD2-expressing neuroblastoma cells [62]. 8B6 mAb induced growth inhibition of *O*AcGD2 positive neuroblastoma cell lines in vitro and apoptosis by caspase 3 activation. A 8B6 mAb treatment also suppressed neuroblastoma tumor growth in mice by reducing the proliferation and increasing apoptosis [93]. Further studies are required to define the molecular mechanisms and signaling pathways involved in anti-*O*AcGD2 mAbs mediated-tumor cell apoptosis.

#### 2.2.2. Biological Roles of Fuc-GM1 in Cancer

Understanding the biological functions of Fuc-GM1 is of great interest to optimize the use of this ganglioside as a potential therapeutic target. Some data suggest that Fuc-GM1 and GM1 are potent inhibitors of PKC activity in PC12 cells [94]. Intracellular signaling pathways regulated by b-series gangliosides such as GD2 and GD3 have been extensively studied in health and diseases. However, almost nothing is known about how Fuc-GM1 regulates the PKC system and other signaling pathways in unique manners, and how this affects cellular biological processes. The role of miR-339-5p was investigated in lung carcinoma cell lines regarding their properties and chemoresistance to Taxol [95]. This miRNA that targets the α1,2-fucosyltransferase *FUT1* is down-regulated in Taxol-treated cells and modulates colony formation, apoptosis, and sensitivity to Taxol in lung carcinoma cell lines, highlighting, again, the role of α1,2-fucosylated-glycoconjugates (most likely Fuc-GM1 and Lewis Y) in SCLC properties.

## 3. Immunotherapy Targeting Cancer-Associated Gangliosides

Cancer immunotherapy corresponds to a variety of treatments using antibodies targeting cancer-associated antigens, such as the different gangliosides species that were the topic of this review.

A phase I clinical trial with anti-GD3 murine IgG3 clone R24 in patients with malignant melanoma demonstrated tumor regression, T-cell activation, and induction of both ADCC and CDC [96]. R24 mAb has been tested in a series of clinical trials in patients with metastatic melanoma and induced responses in some of the patients; importantly, one trial with R24 and interleukin-2 resulted in a higher response rate for review [97]. A vaccine to GD3, named BEC-2, was tested in a phase III trial on patients with SCLC with completed initial chemotherapy [98].

Recently, an anti-GD2 mAb, Dinutuximab (UnituxinTM, ch14.18), has been approved by the Food and Drug Administration (FDA) and the European Medicines Agency (EMA) for the treatment of high-risk pediatric neuroblastoma [99]. A randomized clinical trial demonstrated a significant improvement of patient outcomes (improved event-free survival and overall survival) when Dinutuximab was used in combination with a granulocyte macrophage colony-stimulating factor (GM-CSF), interleukin-2, and 13-cis retinoic acid in the treatment of pediatric patients with high-risk neuroblastoma after a first multiagent/multimodal therapy [100]. Dinutuximab binds to GD2 and induces ADCC and CDC and subsequent NB cell death in a more effective manner than the anti-GD2 14G2a mAb by recruiting granulocytes and natural killer cells from peripheral blood mononuclear population [101].

Multiple clinical trials using different anti-GD2 antibodies have been performed or are currently ongoing in different types of GD2-positive tumors (neuroblastoma, glioblastoma, and lung cancer), most often in combination with other drugs. In SCLC cell lines, the use of anti-GD2 14G2a mAb induced cell death, either by anoikis by FAK dephosphorylation or by apoptosis via *p*38, c-jun terminal kinase and caspase-3 activation [90]. In neuroblastoma cell lines, treatment with the anti-GD2 14G2a mouse mAb inhibited the PI3K/Akt/mTOR pathway and transiently increased PTEN (a suppressor of this pathway), leading to proliferation inhibition [102]. 14G2a mAb also inhibited cell invasion and viability in OS cells, as well as MMP-2 activity. The use of an ETAR (Endothelin-1 (ET-1)/endothelin A receptor, mediating a pathway involved in OS progression) antagonist enhanced the anti-tumor effects of 14G2a mAb through the inhibition of the PI3K/Akt pathway [103].

In addition to anti-GD2 mAb, anti-GD2 therapeutic approaches include disialoganglioside GD2 vaccines [104] and conjugated antibodies, such as immunocytokines, immunotoxins, antibody-drug conjugates, radiolabeled antibodies, targeted nanoparticles, and T-cell engaging bispecific antibodies for review [105]. For example, the coating of nanoparticles with the anti-GD2-ch14.18/CHO mAb allowed the delivery of a specific the aromatase inhibitor, Letrozole, in GBM, inducing reduced proliferation, migration, and chemoresistance in patient-derived GBM tumors [106].

Finally, chimeric antigen receptor T-cell therapy shows extremely promising results in different types of GD2-positive solid tumors. The engineering of new generations of CAR-T cells used in combination with drugs, such PD-1 inhibitors, now allows for their activation, as well as potent immediate-effector functions without evidence of functional exhaustion in vitro in metastatic melanoma [107]. The use of a HGF receptor-neutralizing antibody in combination with GD2-specific CAR-T-cell therapy inhibited Ewing sarcoma (EWS) tumor growth and metastasis in preclinical models [108]. Another study showed the benefit of using bi-functional mesenchymal stem cells (MSC) and expressed high levels of pro-apoptotic agent tumor necrosis factor-related apoptosis-inducing ligand TRAIL and GD2 CART-T for efficient anti-tumor activity in GD2-positive GBM cells [109]. Additionally, the use of GD2-targeted CAR T cells showed promising results in five independent xenograft models of tumor cells from patients with diffuse midline gliomas (DMGs) with mutated histone H3 K27M, which are aggressive and fatal pediatric brain cancers [110].

The use of anti-GD2 mAbs, although largely beneficial for neuroblastoma patients with the use of anti-GD2 mAb Dinutuximab/IL-2/GM-CSF therapy, shows toxic side-effects such as allodynia due to the expression of GD2 on peripheral nerves. The O-acetylated derivative of GD2, OAcGD2, is not expressed on peripheral nerves, and mAb 8B6 could inhibit the growth of OAcGD2-positive tumor cell lines in vitro and could also suppress tumor growth in mice by reducing the proliferation index and inducing the caspase 3-dependent mitochondrial apoptotic pathway [93]. Anti-OAcGD2 mAb could also inhibit glioblastoma cell proliferation in vitro and in vivo [65].

Importantly, the anti-OAcGD2 8B6 mAb showed anti-tumor activity similar to the anti-GD2 14.18 mAb, without inducing allodynia, in a neuroblastoma rat model [62]. The lack of OAcGD2 expression on peripheral nerve fibers and the absence of allodynic properties after c.8B6 treatment provide an important rationale for the clinical application of 8B6 mAb in patients with high-risk neuroblastoma, and possibly in other GD2 and OAcGD2-positive cancers such as glioblastoma, breast cancer, or sarcoma.

The expression of Fuc-GM1 in most SCLC tumors and its absence in normal tissues also makes it an attractive target for cancer immunotherapy. The antitumor activity of BMS-986012, a nonfucosylated fully human IgG1 antibody that binds specifically to Fuc-GM1, was tested in vitro using SCLC cell lines and in mouse xenograft and syngenic tumor models, with and without chemotherapeutic agents and checkpoint inhibitors that are part of the classical treatment for SCLC. These studies demonstrated strong antitumor activity of the BMS-986012 anti-Fuc-GM1 mAb, especially in combination with chemotherapeutic or immunomodulatory agents in these SCLC models [111]. A Phase 1/2 multicenter study of BMS-986012 in subjects with relapsed/refractory SCLC is currently ongoing (ClinicalTrials.gov Identifier: NCT02247349).

Another strategy was used with the engineering of vaccines: in clinical studies, patients with SCLC who were vaccinated with KLH-conjugated Fuc-GM1 developed a serological response with the induction of both IgM and IgG antibodies against Fuc-GM1 [112]. These antibodies showed binding to Fuc-GM1-positive tumor cells and CDC against Fuc-GM1-expressing cell lines in vitro. However, carbohydrate structures were poorly immunogenic and the synthesis of constructs was achieved in order to upgrade the immunogenicity of the candidate carbohydrate-based vaccine [113]. For example, Fuc-GM1 was coupled to a linker (that helped to present the carbohydrate structure to T-cells for activation and initiation of the cellular response) attached to the Keyhole Limpet Hemocyanin (KLH) protein. Another strategy to obtain a broader immune response against a tumor that is often highly heterogenous is to combine the synthetic Fuc-GM1 vaccine with vaccines against three other carbohydrate antigens highly expressed in SCLC: GM2, Globo H, and polysialic acid [114].

## 4. Conclusions

Gangliosides are promising targets for cancer immunotherapy, especially in combination with drugs. Nevertheless, for each tumor type, ganglioside composition has a great impact on treatment efficiency, which has to be adapted to the specific molecular pattern of the tumor. In particular, the aberrant expression of Fuc-GM1 in a restricted variety of tumors and the role of this glycolipid in cancer remain to be elucidated. Lung and hepatocellular carcinoma can be considered as neuro-endocrine derived-tumors, and Fuc-GM1 could be a hallmark of such tumors, whereas GD3, GD2, and their O-acetylated derivatives are hallmarks of neuroectoderm-derived cancers. Particular efforts have to be continued to understand the molecular mechanisms underlying the over expression of gangliosides in specific tumor types, especially the multi-level regulation of key Golgi-localized glycosyltransferases (GD3 synthase, GD2 synthase, *FUT1*, *FUT2*, *CASD1*), and their effects of synthesized gangliosides on the malignant properties of cancer cells (activated signaling pathways, gene/protein networks involved) to optimize therapeutic strategies.

## Data Availability

Not applicable.
